# Habitat selection by the European hare in arable landscapes: The importance of small‐scale habitat structure for conservation

**DOI:** 10.1002/ece3.4613

**Published:** 2018-11-13

**Authors:** Martin Mayer, Wiebke Ullmann, Peter Sunde, Christina Fischer, Niels Blaum

**Affiliations:** ^1^ Department of Bioscience Aarhus University Rønde Denmark; ^2^ Plant Ecology and Conservation Biology University of Potsdam Potsdam Germany; ^3^ Institute for Landscape Biogeochemistry Leibniz‐Centre for Agricultural Landscape Research (ZALF) Müncheberg Germany; ^4^ Restoration Ecology, Department of Ecology and Ecosystem Management Technische Universität München Freising Germany

**Keywords:** agriculture, arable land, conservation, GPS, habitat selection, *Lepus europaeus*, vegetation height

## Abstract

Agricultural land‐use practices have intensified over the last decades, leading to population declines of various farmland species, including the European hare (*Lepus europaeus*). In many European countries, arable fields dominate agricultural landscapes. Compared to pastures, arable land is highly variable, resulting in a large spatial variation of food and cover for wildlife over the course of the year, which potentially affects habitat selection by hares. Here, we investigated within‐home‐range habitat selection by hares in arable areas in Denmark and Germany to identify habitat requirements for their conservation. We hypothesized that hare habitat selection would depend on local habitat structure, that is, vegetation height, but also on agricultural field size, vegetation type, and proximity to field edges. Active hares generally selected for short vegetation (1–25 cm) and avoided higher vegetation and bare ground, especially when fields were comparatively larger. Vegetation >50 cm potentially restricts hares from entering parts of their home range and does not provide good forage, the latter also being the case on bare ground. The vegetation type was important for habitat selection by inactive hares, with fabaceae, fallow, and maize being selected for, potentially providing both cover and forage. Our results indicate that patches of shorter vegetation could improve the forage quality and habitat accessibility for hares, especially in areas with large monocultures. Thus, policymakers should aim to increase areas with short vegetation throughout the year. Further, permanent set‐asides, like fallow and wildflower areas, would provide year‐round cover for inactive hares. Finally, the reduction in field sizes would increase the density of field margins, and farming different crop types within small areas could improve the habitat for hares and other farmland species.

## 1 INTRODUCTION

Agricultural landscapes dominate in large parts of the world, with 38% of the Earth's ice‐free surface being covered by cropland and pasture (Foley et al., 2011). In Europe, pastures (permanent grassland and meadow) cover 14.4% of the land area and arable land (cropland used under a system of crop rotation) accounts for 26.5% of the area, making Europe one of the most intensely used agricultural areas (Ramankutty, Evan, Monfreda, & Foley, 2008). Accordingly, agricultural areas are important habitats for a wide range of Europe's biodiversity, including birds and mammals of which some have adapted to these culturally influenced habitats. Since the beginning of the 20th century, agriculture intensified steadily in Europe, leading to increased yields due to larger field sizes, the use of agro‐chemicals, and the improved efficiency of machinery (Marshall & Moonen, 2002; O'Brien & De La Escosura, 1992; Smith, Jennings, Robinson, & Harris, 2004). This intensification ultimately led to a decreased habitat heterogeneity (Benton, Vickery, & Wilson, 2003), causing a steep decline in biodiversity (Reidsma, Tekelenburg, Berg, & Alkemade, 2006), for example, abundance and species richness of plant species (Storkey, Meyer, Still, & Leuschner, 2011) and farmland birds (Bowler, Heldbjerg, Fox, O'Hara, & Böhning‐Gaese, 2018; Donald, Green, & Heath, 2001; Heldbjerg, Sunde, & Fox, 2017).

**Figure 1:**
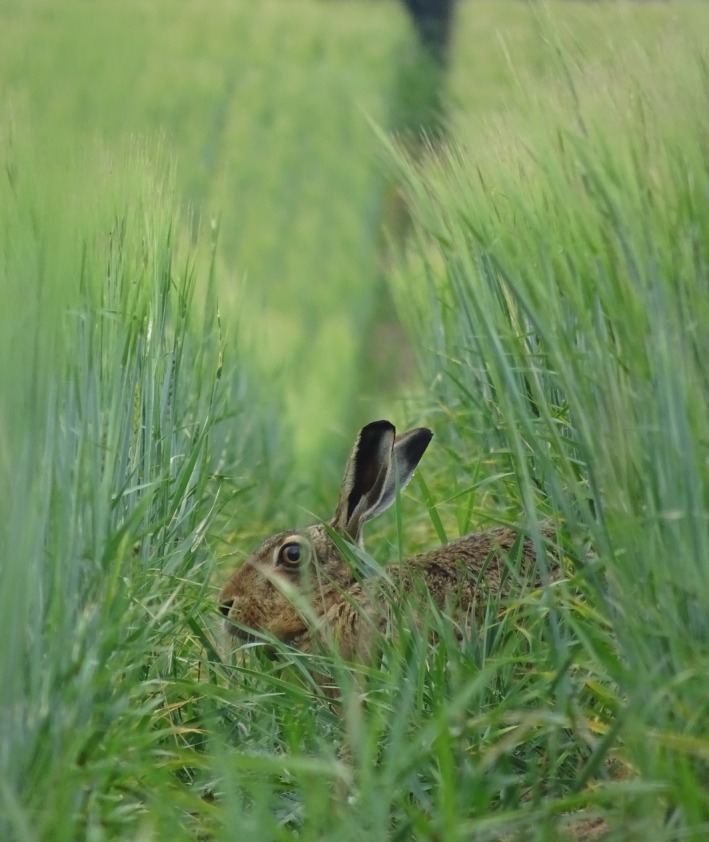
Our study species, the European hare (*Lepus europaeus*) in a barley field in Denmark

Agricultural land is the main habitat of the European hare (*Lepus europaeus*, hereafter hare, Figure 1) (Frylestam, 1980; Vaughan, Lucas, Harris, & White, 2003). Hares have declined throughout Europe since 1960 (Smith, Jennings, & Harris, 2005) and are classified as “near threatened” or “threatened” on the Red List of Threatened Species in several countries, for example, Austria, Germany, Norway, and Switzerland (Boye, 1996; Reichlin, Klansek, & Hackländer, 2006). There is an increasing body of literature suggesting that agricultural intensification is the ultimate reason for the decline in hare populations (Smith et al., 2005 and references therein), although predation, disease, hunting, and a changing climate may also be population‐limiting factors (Edwards, Fletcher, & Berny, 2000; Hackländer, Arnold, & Ruf, 2002; Lindström et al., 1994). Hence, in order to implement effective conservation measures, it is important to investigate the elements affecting hare habitat use in intensively used agricultural landscapes.

Home range sizes of hares increase with agricultural field size, and hares generally select for proximity to field edges (Petrovan, Ward, & Wheeler, 2013; Schai‐Braun & Hackländer, 2013) and avoid roads (Roedenbeck & Voser, 2008). Moreover, it was shown that hares utilize different habitats when being active (typically during nighttime) for foraging compared to when resting (typically during daytime) (Neumann, Schai‐Braun, Weber, & Amrhein, 2012; Tapper & Barnes, 1986). However, little is known about how habitat and vegetation structure affects within‐home‐range habitat selection in arable landscapes (but see Tapper & Barnes, 1986). Smith et al. (2004) investigated how vegetation height affected habitat selection by hares in pastural landscapes in Britain. They argued that there is a greater potential to increase hare numbers in pastural landscapes compared to arable land, because in pastural landscapes, hare densities are comparatively lower and hares are in poorer body condition. In the United Kingdom, 63% of the agricultural land is pastural, and 37% is arable land. However, in most western (apart from Great Britain), central, and northern European countries, arable land makes up the majority of the agricultural landscape (Table 1). For example, arable land accounts for 71% of the agricultural used land in Germany and for 92% in Denmark (Table 1). Thus, for large parts of Europe, arable land is highly important for hares simply because it makes up such a large proportion of its habitat.

**Table 1:** The percentage of land‐use type in selected European countries in 2013 (Source: https://ec.europa.eu/eurostat/statistics-explained/index.php/Farm_structure_statistics#Agricultural_land_use)

Country	Land‐use type		
	Arable land	Pastural land	Other
Denmark	91.5	7.5	1.02
Sweden	85.1	14.8	0.16
Hungary	81.6	15.1	3.29
Poland	74.7	22.3	3.08
Slovakia	71.7	27.3	1.04
Czech Republic	71.4	27.5	1.13
Germany	71.1	27.7	1.21
Bulgaria	70.5	27.3	2.16
France	66.6	29.7	3.72
Belgium	61.1	37.2	1.67
Netherlands	56.2	41.8	1.98
Croatia	55.9	39.3	4.75
Austria	50.0	47.5	2.45
Luxembourg	47.8	51.1	1.18
United Kingdom	36.7	63.1	0.21
Slovenia	35.6	58.6	5.82
Ireland	21.0	79.0	0.03
European Union	59.8	34.2	6.1

In this study, we investigated within‐home‐range habitat selection by hares in agricultural landscapes dominated by arable land in Denmark and Germany using GPS technology. Arable crops greatly change both within and between the vegetative seasons, providing cover and food during parts of the year, but not during others when high crops potentially represent a barrier and decrease forage quality, and plowed fields restrict cover and forage. Further, the size of agricultural fields should be important, because areas with larger fields are more homogenous, providing less cover and foraging opportunities (Petrovan et al., 2013; Schai‐Braun & Hackländer, 2013), resulting in increased home range sizes (Ullmann, Fischer, Pirhofer‐Walzl, Kramer‐Schadt, & Blaum, 2018). Thus, we hypothesized that both vegetation height and field size would be more important in explaining habitat selection by hares than the vegetation type itself. This is important, because using a measure of vegetation height and field size rather than crop types would facilitate the identification of vital habitat requirements for hares and other threatened farmland species, in turn providing simple guidelines to increase the habitat quality. We calculated hare home range sizes to investigate the influence of field size, vegetation height, and vegetation type on hare habitat selection. Specifically, we predicted that hares would select for comparatively shorter vegetation when being active as this provides better forage and allows the detection of predators, and for comparatively higher vegetation when inactive (providing cover). Similarly, we predicted that active hares would select for vegetation types that provide good forage (e.g., fallow, pasture, young cereals) and inactive hares select for vegetation types that provide good cover (e.g., fabaceae, maize). Further, we predicted that hares would generally select for smaller fields, because they constitute a more heterogeneous landscape, and more so with increasing vegetation height, because high vegetation potentially represents a barrier to enter further into (larger) fields. Finally, we predicted that hares would select for proximity to field edges, because they increase habitat heterogeneity (Petrovan et al., 2013) providing both cover and food, and more so with increasing vegetation height, because high vegetation might represent a physical barrier.

## 2 MATERIALS AND METHODS

### 2.1 Study area

**Figure 2:**
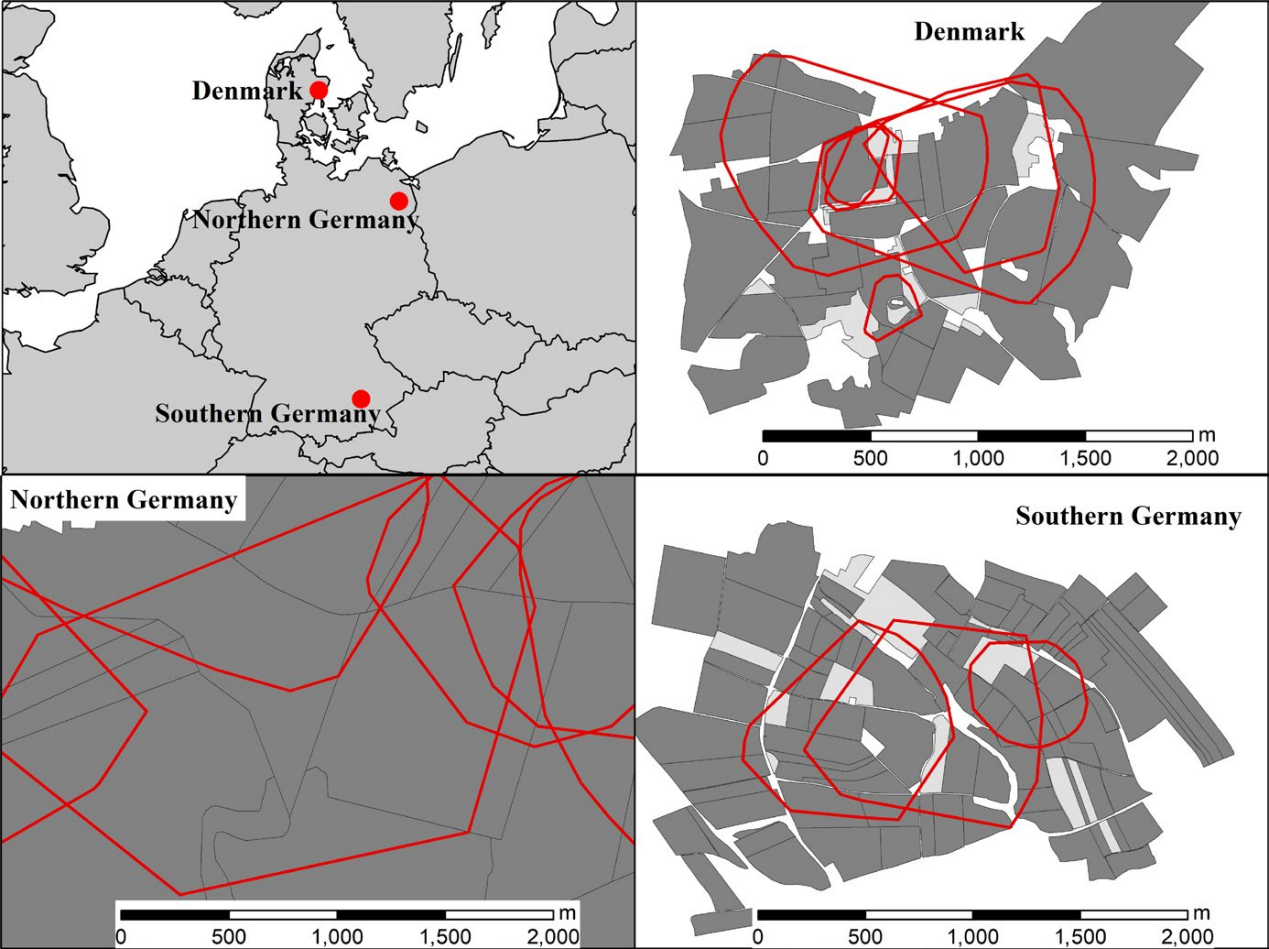
Map showing the location of the three study areas (red dots, top left), and exemplary European hare (*Lepus europaeus*) home ranges (red lines) from Denmark (top right), northern Germany (bottom left), and southern Germany (bottom right). Arable fields are shown in dark gray, pastures in light gray. Hare GPS data were obtained in 2014–2015

We conducted fieldwork in three study areas that were located in (a) Syddjurs community, Midtjylland region, Denmark (hereafter Denmark), (b) Uckermark, Brandenburg, Germany (hereafter northern Germany), and (c) Freising, Bavaria, Germany (hereafter southern Germany) (Figure 2). The landscape was dominated by arable land in all three study areas. The Danish study area mostly consisted of arable fields (94%) tilled with wheat (*Triticum aestivum*), barley (*Hordeum vulgare*), rapeseed (*Brassica napus*), beans (*Vicia faba*), and oats (*Avena sativa*). The rest of the area consisted of meadow, game fields, and fallow. The study area in northern Germany primarily consisted of large arable fields (90%) interspersed with some forest patches, pastures, urban areas, and water (InVeKoS, 2014). Wheat, barley, rapeseed, and maize (*Zea mays*) were the dominant crop types, but sugar beet (*Beta vulgaris*), charlock mustard (*Sinapis arvensis*), and triticale were also present. The study area in southern Germany mostly consisted of smaller arable fields (83%) interspersed with forest patches, pastures, water, and urban areas (Vermessungsverwaltung, 2014). Wheat, maize, barley, rapeseed, and charlock mustard were the most common crop types, but hops (*Humulus lupulus*), pastures, sugar beet, rye (*Secale cereale*), triticale, clover (*Trifolium* spp.), oats, peas (*Pisum sativum*), and potatoes (*Solanum tuberosum*) were also cultivated. Hare density in both German areas was approximately 5 hares per km^2^, but fox density was higher in northern Germany (ca. 0.8 per km^2^) than in southern Germany (ca. 0.2 per km^2^; Wiebke Ullmann, unpublished results). We did not obtain data on hare and fox densities in Denmark.

### 2.2 Hare captures

In Denmark, we captured hares in 2014 using box traps that were set up in pairs along the edges of agricultural fields. In Germany, we captured hares in 2014 and 2015 by driving them into nets (Rühe & Hohmann, 2004). We transferred captured hares into a canvas cone (Denmark) or a wooden box (Germany), where they could be handled without anesthesia. Hares were sexed and fitted with a GPS collar (e‐obs A1, e‐obs GmbH, Gruenwald, Germany). GPSs in Denmark were set to take one‐hourly GPS positions. In the two German areas, GPSs were set to take one‐hourly positions while hares were active (defined by an acceleration threshold), and to take four‐hourly positions when hares were inactive. We obtained GPS data from May until December in Denmark, from May until January (the following year) in southern Germany, and from all months in northern Germany.

### 2.3 Data preparation

#### 2.3.1 Habitat data

We categorized the different crop species in the variable “vegetation type,” consisting of 11 categories based on biological knowledge (Table 2). Other landscape elements (e.g., forest, permanent plantations, and water banks) were excluded, because they made up a negligible proportion of individual hare home ranges (<1%). The variable “vegetation height” was grouped into five categories: no vegetation (bare ground), 1–25 cm, >25–50 cm, >50–100 cm, and >100 cm. We used this categorization, because vegetation height was measured too infrequently (monthly or bimonthly depending study area and year), and because crops grow very fast during the vegetative season, not allowing for a precise continuous variable. In Denmark, the height category “>100 cm” was absent, because crops did not grow over 100 cm in height. We calculated the size of agricultural fields (in ha) in ArcMap 10.4.1 (Esri, Redlands, CA, USA), defined as the continuous variable “field size.” Further, we calculated the Euclidean distance of GPS positions to field edges as a measure of proximity to field edges in ArcMap, defined as the continuous variable “edge distance.”

**Table 2:** Showing the crop species and agricultural treatments that we categorized into the 12 different vegetation types, and the number of random and used GPS positions. Percentages of random and used GPS positions are given in parentheses

Vegetation type	Crop species/agricultural treatment	Random GPS positions	Used GPS positions
Beet	Sugar beet (*Beta vulgaris*)	1,860 (43.6)	2,409 (56.4)
Brassicaceae	Charlock mustard (*Sinapis arvensis*), rapeseed (*Brassica napus*), winterrape	6,794 (57)	5,118 (43)
Cereal	Barley (*Hordeum vulgare*), oats (*Avena sativa*), rye (*Secale cereale*), triticale, wheat (*Triticum aestivum*), winterbarley, winterwheat	25,600 (54.2)	21,612 (45.8)
Fabaceae	Beans (*Vicia faba*), peas (*Pisum sativum*)	2,113 (38.3)	3,408 (61.7)
Fallow	Fallow and game fields consisting of various plant species	2,035 (41.9)	2,822 (58.1)
Fodder	Agricultural grass, clover (*Trifolium* spp.)	1,021 (53.1)	901 (46.9)
Hops	Hops (*Humulus lupulus*)	381 (50.3)	377 (49.7)
Maize	Maize (*Zea mays*)	12,529 (49.2)	12,957 (50.8)
No vegetation (bare ground)	Harrowed, plowed, raked, and freshly sown ground	10,246 (45.5)	12,261 (54.5)
Pasture	Meadow and pasture	11,233 (42.5)	15,222 (57.5)
Stubbles	Harvested cereal, maize, and rape	5,529 (46.2)	6,446 (53.8)

#### 2.3.2 GPS data

We removed individuals, where the GPS failed after a short time period (<100 GPS positions; 12 of 64 individuals). Further, we removed the first day from the analysis to avoid possible effects of capture and handling. We then calculated the home range size of individual hares based on 95% minimum convex polygons (MCP) during each individuals’ sampling period (mean ± *SD*: 1,607 ± 1,157 individual GPS positions) in R 3.2.5 (R Core Team, 2013) using the adehabitatHR package (Calenge, 2006). We used MCPs instead of kernel density estimation, because the latter potentially excludes available but unused areas, which could bias the analysis. To get a measure of resource availability, we created the same number of random GPS positions than we had obtained from each hare within each individual hare home range. We then assigned each random and used (hare) GPS position to the vegetation type, vegetation height, field size, and the edge distance using the “join” tool in ArcMap. We removed all GPS positions (both used and random) that could not be assigned to a vegetation type or height (14% of the data). To obtain a proxy of activity, we calculated the straight‐line distance between consecutive (i.e., hourly) hare GPS positions (Schai‐Braun, Rödel, & Hackländer, 2012). We then plotted the average distance moved per hour against the time of the day separately for long (>12 hr daylight) and short (<12 hr daylight) days, because hares shift their activity with changing daylight length (Schai‐Braun et al., 2012). Further, we plotted them separately for the three study areas, because hare home range sizes differed significantly between areas (see Results), leading to different hourly movement distances (Supporting Information Figure S1). Finally, we calculated the overall average distance moved (separately for the three areas), and set the threshold for activity as 75% of the overall average distance moved, that is, we categorized hares as “active” if hourly distance moved was >75% of the average distance moved, and “inactive” if it was <75% of the average distance moved (Supporting Information Figure S1).

### 2.4 Statistical analysis

We used resource selection functions (Manly, McDonald, Thomas, McDonald, & Erickson, 2007) to investigate within‐home‐range habitat selection by hares separately for active and inactive GPS positions due to different habitat requirement for foraging and resting (Neumann et al., 2012). We built generalized linear mixed models (GLMM) with a Bernoulli distribution and a logit link as dependent variable (1 = used (hare) GPS position versus 0 = available (random) GPS position). To investigate the relative importance of field and vegetation features for habitat selection, we created four candidate models, including one fixed effect per model: (a) vegetation type, (b) vegetation height, (c) field size, and (d) the quadratic function of edge distance (fitted better than the linear function based on Akaike's information criterion corrected for small sample size (AIC_c_) (Burnham, Anderson, & Huyvaert, 2011)). The vegetation type “cereal” and the vegetation height “>25–50 cm” were used as reference, because they were present and largely available in all study areas. The hare ID, area, and month nested within year (to control for seasonal and annual effects) were included as random intercept.

We then investigated finer‐scale habitat selection using GLMMs (1 = used, 0 = available) separately for active and inactive GPS positions. Fixed effects were the vegetation type, vegetation height, field size, and the quadratic function of the edge distance. We included two interactions: (a) vegetation height × edge distance to test whether hares would select for proximity to field edges with increasing vegetation height and (b) vegetation height × field size to investigate whether higher vegetation was a greater barrier in larger fields. For this analysis, we merged vegetation heights “>50–100 cm” and “>100 cm,” because vegetation >50 cm was generally avoided (see results). Hare ID, area, and month nested within year were included as random intercept to control for annual/seasonal variation and multiple observations. After initially checking for sex differences in habitat selection, we did not include this variable in our main analyses, because we found no differences between females and males. We used a set of 20 candidate models including different combinations of the fixed effects and the above‐described interactions (Supporting Information Table S1).

Field size and edge distance were log‐transformed to normalize residuals of the statistical models. We found no collinearity among fixed effects (*r* < 0.6 in all cases), and variance inflation factors were <3 (Zuur, Ieno, & Elphick, 2010). Model selection was based on AIC_c_ and AIC weights (Burnham et al., 2011) and was carried out using the R package MuMIn (Barton, 2013). If ∆AIC_c_ was <10 in two or more of the most parsimonious models, we performed model averaging (Anderson, 2008; Bolker et al., 2009). Parameters that included zero within their 95% CI were considered uninformative (Arnold, 2010). We validated the most parsimonious models by plotting the model residuals versus the fitted values (Zuur, Ieno, Walker, Saveliev, & Smith, 2009). All statistical analyses were carried out in R 3.2.5 (R Core Team, 2013).

## 3 RESULTS

### 3.1 Home range sizes and agricultural field sizes

We obtained data of 52 individuals (28 in northern Germany, 18 in southern Germany, and 6 in Denmark), 22 females and 30 males, from which we got 1,607 ± 1,157 (mean ± *SD*) individual GPS positions, resulting in a total of 83,533 GPS positions (61,746 active and 21,787 inactive positions) that we could assign to different habitat parameters. Individual home range sizes varied between 4 and 150 ha. After controlling for different GPS sampling durations (by including the number of individual GPS locations), home ranges were significantly larger in northern Germany (77 ± 43 ha) compared to Denmark (44 ± 41 ha) and southern Germany (30 ± 19 ha, linear regression: *p* < 0.01). Home range sizes in Denmark did not differ significantly from southern Germany (*p* = 0.54). Further, agricultural fields in northern Germany were significantly larger compared to southern Germany and Denmark (*t* test: *t* > 6, *df *> 78, *p* < 0.001), and Danish fields were significantly larger compared to southern Germany (*t* = 2.31, *df* = 48.1, *p* = 0.03).

### 3.2 Habitat selection

#### 3.2.1 Relative importance of habitat type and structure

When evaluating the relative importance of habitat type and structure for habitat selection by hares, the model including the vegetation height was by far the best (AIC weight = 1) for active GPS positions, followed by vegetation type, field size, and edge distance (Table 3). When investigating inactive GPS positions, the model including vegetation type was the best (AIC weight = 1), followed by vegetation height, field size, and edge distance (Table 3).

**Table 3:** The model selection result for the candidate models investigating the relative importance of habitat type and habitat structure for habitat selection by European hares (*Lepus europaeus*) based on data collected in Denmark and Germany (2014–2015). Hare ID, area, and month were included as random effects. Models were ranked based on AIC_c_

Model	*df*	logLik	AIC_c_	Delta AIC_c_	AIC_c_ weight
Active hare GPS positions					
Vegetation height	9	−82,128	164,275	0	1
Vegetation type	15	−82,619	165,268	993	0
log (field size)	6	−83,096	166,205	1,930	0
log (edge distance) + log (edge distance)^2	7	−83,165	166,344	2,069	0
Inactive hare GPS positions					
Vegetation type	15	−28,554	57,139	0	1
Vegetation height	9	−28,831	57,681	542	0
log (field size)	6	−29,143	58,298	1,159	0
log (edge distance) + log (edge distance)^2	7	−29,160	58,335	1,196	0

#### 3.2.2 Active GPS positions

**Figure 3:**
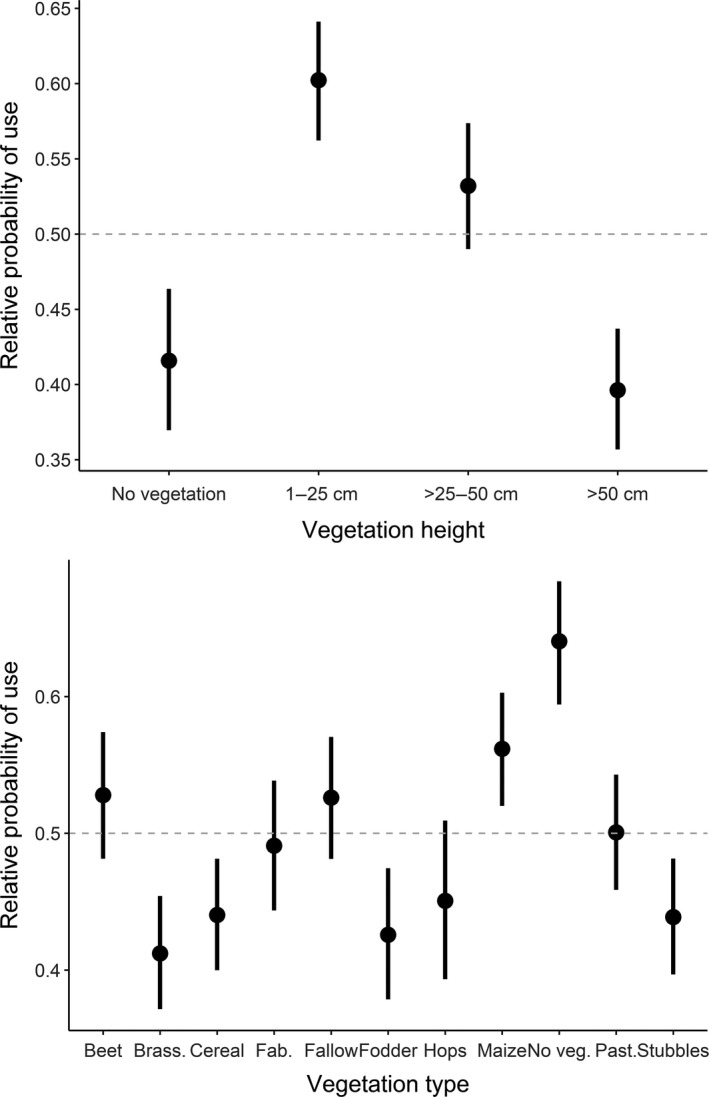
The effect of vegetation height (top) and vegetation type (bottom) on the relative probability of use by active European hares (*Lepus europaeus*). Values >0.5 indicate selection, whereas values <0.5 indicate avoidance. The 95% confidence intervals are given as bars. Data were obtained from 52 GPS‐collared hares in Denmark and Germany (2014–2015). Brass. = brassicaceae, Fab. = fabaceae, No veg. = no vegetation, Past. = pasture

**Figure 4:**
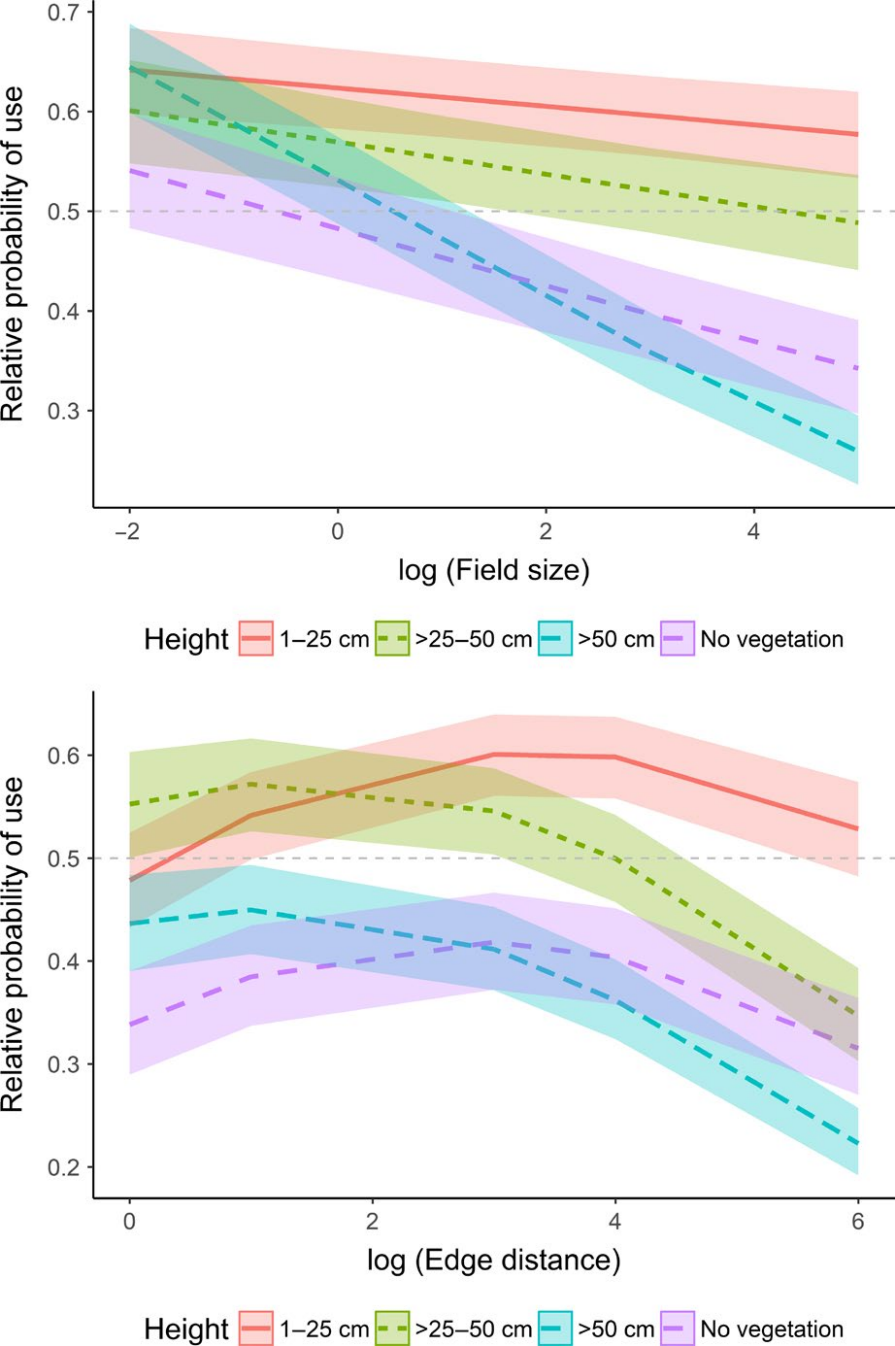
Effect plots showing the effect of the interaction between vegetation height and field size (log‐transformed; top), and between vegetation height and edge distance (log‐transformed; bottom) on the relative probability of use by active European hares (*Lepus europaeus*). Values >0.5 indicate selection, whereas values <0.5 indicate avoidance. The 95% confidence intervals are given as shading. Data were obtained from 52 GPS‐collared hares in Denmark and Germany (2014–2015)

When investigating finer‐scale habitat selection, the full model performed best in explaining habitat selection by active hares (Table 4 and Supporting Information Table S1). With >25–50 cm high vegetation as reference, active hares had a higher relative probability (hereafter referred to as “selection”) to use short vegetation (1–25 cm) and a lower relative probability (hereafter referred to as “avoidance”) to use higher vegetation (>50 cm) and bare ground (Table 4, Figure 3). There was no apparent selection for or against >25–50 cm high vegetation (Figure 1). Concerning the vegetation type and with cereals as reference, active hares selected for bare ground, fabaceae, sugar beet, fallow, maize, and pasture, and avoided brassicaceae (Table 4). There was no apparent selection for or against fodder, hops, and stubbles. Relative to random locations, we found that active hares generally selected for bare ground and maize, avoided brassicaceae, cereal, fodder, and stubbles, and showed no apparent selection for or against sugar beet, fabaceae, fallow, hops, and pasture (Figure 3). Further, the interaction between vegetation height and field size showed that active hares generally selected for shorter vegetation (1–50 cm) and avoided vegetation >50 cm and bare ground with increasing field sizes (Figure 4). When field sizes were smaller (in southern Germany), there was no apparent selection for or against a specific vegetation height (CIs overlapped; Figure 4). The interaction between vegetation height and edge distance revealed that active hares selected for proximity to field edges when vegetation height was >25 cm, but selected for intermediate distances from field edges in short vegetation (1–25 cm) and on bare ground (Figure 4).

**Table 4:** Effect size (β), standard error (*SE*), lower 95% confidence interval (LCI) and upper 95% confidence interval (UCI) of explanatory variables for the analyses of habitat selection by European hares in Denmark, southern, and northern Germany (2014–2015) separately for active and inactive hare GPS positions. Informative parameters are given in bold. Positive β values indicate a higher relative probability of use (selection), whereas negative values indicate a lower relative probability of use (avoidance)

Variable	Active hare GPS positions				Inactive hare GPS positions			
	β	*SE*	LCI	UCI	β	*SE*	LCI	UCI
(Intercept)	0.11	0.11	−0.11	0.32	0.26	0.14	−0.01	0.54
Vegetation type no vegetation	**0.82**	**0.07**	**0.69**	**0.95**	**0.39**	**0.11**	**0.17**	**0.61**
Vegetation type fabaceae	**0.20**	**0.05**	**0.10**	**0.31**	**1.37**	**0.06**	**1.26**	**1.49**
Vegetation type beet	**0.35**	**0.05**	**0.26**	**0.44**	**−0.21**	**0.08**	**−0.37**	**−0.05**
Vegetation type brassicaceae	**−0.11**	**0.03**	**−0.17**	**−0.06**	**0.19**	**0.05**	**0.10**	**0.28**
Vegetation type fallow	**0.34**	**0.04**	**0.27**	**0.42**	**0.73**	**0.07**	**0.60**	**0.86**
Vegetation type fodder	−0.06	0.06	−0.17	0.05	**−0.55**	**0.11**	**−0.76**	**−0.33**
Vegetation type hops	0.04	0.09	−0.13	0.21	−0.08	0.17	−0.42	0.25
Vegetation type maize	**0.49**	**0.02**	**0.45**	**0.53**	**0.82**	**0.04**	**0.75**	**0.90**
Vegetation type pasture	**0.24**	**0.02**	**0.20**	**0.29**	**0.27**	**0.04**	**0.19**	**0.35**
Vegetation type stubbles	−0.01	0.03	−0.06	0.05	**0.21**	**0.05**	**0.12**	**0.30**
Vegetation height no vegetation	**−0.76**	**0.10**	**−0.96**	**−0.57**	**−0.55**	**0.18**	**−0.90**	**−0.20**
Vegetation height 1–25 cm	**−0.36**	**0.07**	**−0.49**	**−0.23**	**−0.66**	**0.11**	**−0.88**	**−0.44**
Vegetation height >50 cm	−0.07	0.07	−0.21	0.07	**−0.56**	**0.12**	**−0.80**	**−0.32**
log (edge distance)	**0.12**	**0.03**	**0.07**	**0.18**	**−0.24**	**0.05**	**−0.34**	**−0.14**
log (edge distance)^2	**−0.04**	**0.00**	**−0.05**	**−0.04**	0.01	0.01	−0.01	0.02
log (field size)	**−0.07**	**0.02**	**−0.10**	**−0.03**	**0.08**	**0.03**	**0.02**	**0.14**
Vegetation height no vegetation × log (field size)	**−0.05**	**0.02**	**−0.09**	**−0.01**	**−0.12**	**0.04**	−**0.20**	−**0.04**
Vegetation height 1–25 cm × log (field size)	0.03	0.02	−0.01	0.06	−**0.16**	**0.03**	−**0.23**	−**0.09**
Vegetation height >50 cm × log (field size)	−**0.17**	**0.02**	−**0.21**	−**0.13**	−**0.20**	**0.03**	−**0.27**	−**0.13**
Vegetation height no vegetation × log (edge distance)	**0.12**	**0.02**	**0.08**	**0.17**	**0.23**	**0.04**	**0.15**	**0.31**
Vegetation height 1–25 cm × log (edge distance)	**0.17**	**0.02**	**0.14**	**0.21**	**0.33**	**0.03**	**0.26**	**0.39**
Vegetation height >50 cm × log (edge distance)	−0.02	0.02	−0.06	0.01	**0.15**	**0.03**	**0.08**	**0.22**

#### 3.2.3 Inactive GPS positions

**Figure 5:**
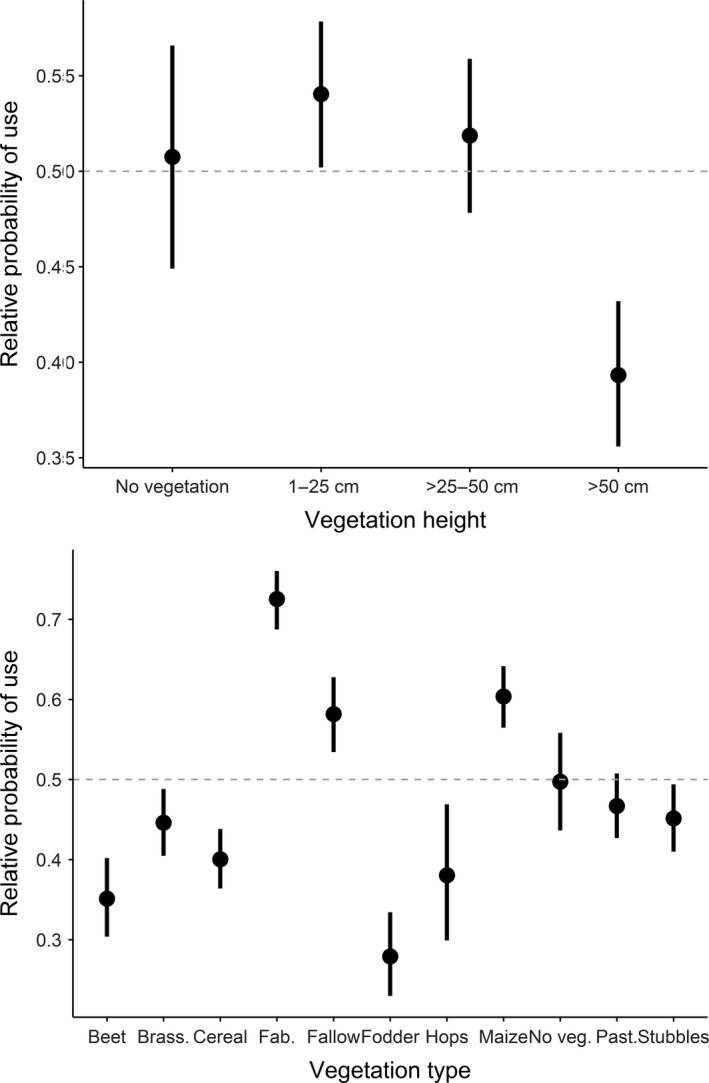
The effect of vegetation height (top) and vegetation type (bottom) on the relative probability of use by inactive European hares (*Lepus europaeus*). Values >0.5 indicate selection, whereas values <0.5 indicate avoidance. The 95% confidence intervals are given as bars. Data were obtained from 52 GPS‐collared hares in Denmark and Germany (2014–2015)

**Figure 6:**
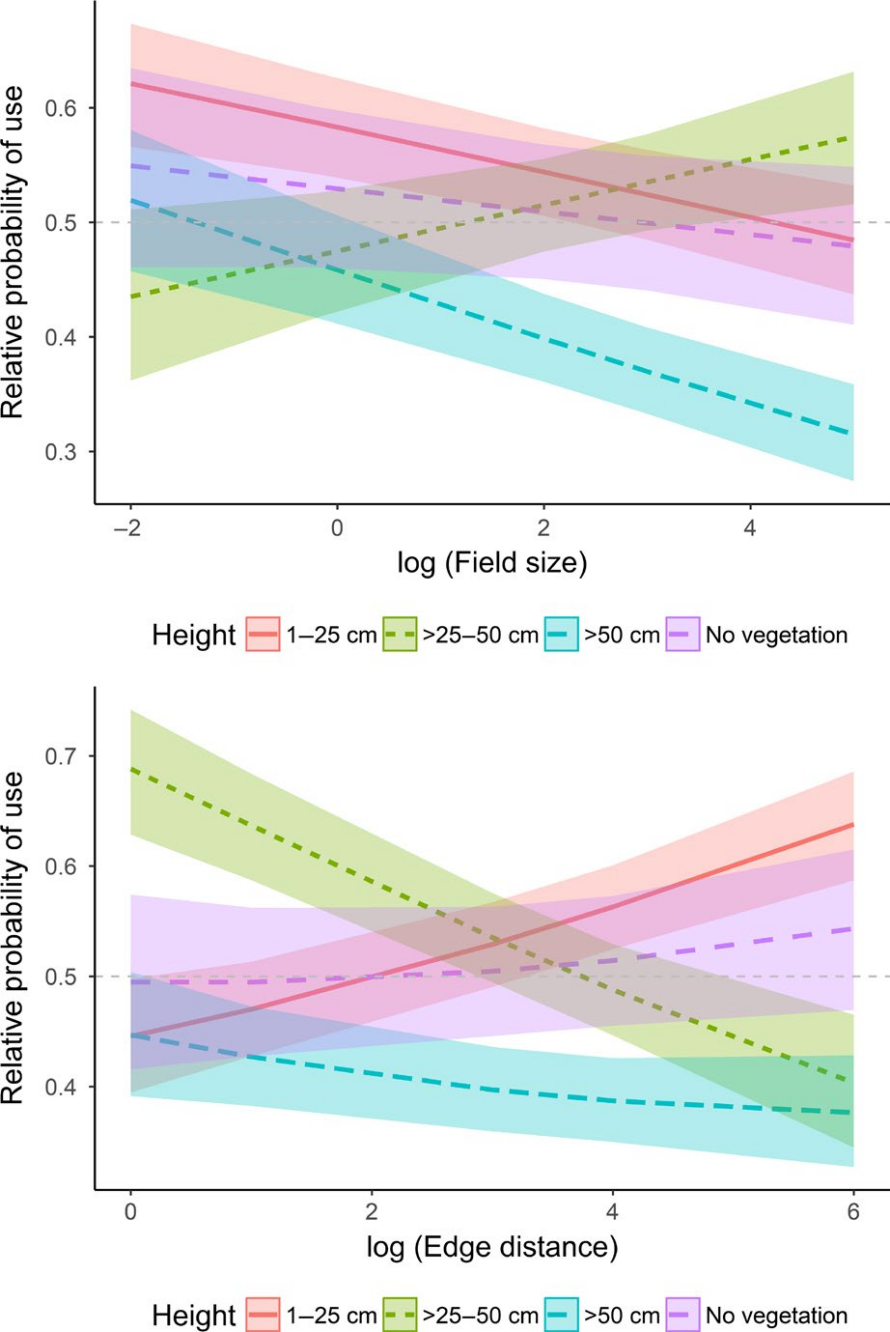
Effect plots showing the effect of the interaction between vegetation height and field size (log‐transformed; top), and between vegetation height and edge distance (log‐transformed; bottom) on the relative probability of use by inactive European hares (*Lepus europaeus*). Values >0.5 indicate selection, whereas values <0.5 indicate avoidance. The 95% confidence intervals are given as shading. Data were obtained from 52 GPS‐collared hares in Denmark and Germany (2014–2015)

Habitat selection analyzed for inactive hare GPS positions was also best explained by the full model (Table 4 and Supporting Information Table S1). With >25–50 cm high vegetation as reference, inactive hares also selected for short vegetation (1–25 cm) and avoided vegetation >50 cm (Table 4, Figure 5). There was no apparent selection for or against >25–50 cm high vegetation and bare ground (Figure 5). Concerning the vegetation type and with cereals as reference, inactive hares selected for bare ground, fabaceae, brassicaceae, fallow, maize, pasture, and stubbles, and avoided sugar beet and fodder (Table 4). There was no apparent selection for or against hops. Relative to random locations, we found that hares generally selected for fabaceae, fallow, and maize, avoided brassicaceae, cereal, fodder, hops, stubbles, and sugar beet, and showed no apparent selection for or against bare ground and pasture (Figure 5). The interaction between vegetation height and field size indicated that with increasing field size, inactive hares selected for >25–50 cm high vegetation and avoided lower and higher vegetation including areas without vegetation (Figure 6). Finally, the interaction vegetation height and edge distance revealed that inactive hares selected for proximity to field edges when vegetation was >25–50 cm high (and to a lesser degree >50 cm) and remained further from field edges in short vegetation (<25 cm) and to a lesser degree on bare ground (Figure 6).

## 4 DISCUSSION

Vegetation height and type, field size, and proximity to field edges all were important in explaining within‐home‐range habitat selection by hares, emphasizing the importance of small‐scale habitat structure in highly variable arable landscapes. Vegetation height was most important for habitat selection of active hares, with short vegetation (1–25 cm) being preferred, possibly for reasons of food quality and predator detection/avoidance. Vegetation type was most important for habitat selection by inactive hares, with fabaceae, fallow, and maize being preferred, potentially providing cover from predators and forage at the same time. Our results also emphasize that differences in field sizes ultimately affect habitat selection by hares.

### 4.1 The role of vegetation height

Both active and inactive hares generally selected for short vegetation (1–25 cm) and avoided vegetation >50 cm. However, selection for specific vegetation height was related to agricultural field sizes and proximity to field edges. Hares avoided higher vegetation, likely because it did not provide good forage, acted as a physical barrier (Rühe, 1999), and impeded their ability to detect predators (Hewson, 1977).

### 4.2 Vegetation height and forage quality

Although hares select for wild weeds during spring and summer, the majority of their diet consists of agricultural crops, because crops dominate the available plant species in arable landscapes throughout the year (Reichlin et al., 2006; Schai‐Braun et al., 2015), a pattern that is increasing with the increasing use of pesticides and fertilizers (Storkey et al., 2011). The amount of standing dead plant biomass increases with increasing height of the standing crop (van de Koppel, Huisman, Wal, & Olff, 1996), leading to a higher proportion of fiber and subsequently to a lower forage quality (Wilmshurst, Fryxell, & Hudsonb, 1995). Thus, it is plausible that active hares avoided higher crops for reasons of decreased forage quality (Tapper & Barnes, 1986) and due to increasingly dense vegetation that could not be accessed (van de Koppel et al., 1996). In addition, active hares avoided areas without any vegetation, likely because bare ground does not provide forage.

### 4.3 Vegetation height and agricultural field size can act as a barrier

Active hares generally selected for short (1–25 cm) vegetation independent of the agricultural field size. Conversely, bare ground and >50 cm high vegetation were increasingly avoided with increasing field size. Similarly, inactive hares avoided >50 cm high vegetation with increasing field size, and both active and inactive hares stayed close to field edges when vegetation was >25 cm high, but not in lower/no vegetation. Combined, the results indicate that larger fields with high and dense vegetation (e.g., brassicaceae, cereals, and maize) potentially presented a physical barrier inhibiting hares from entering farther into them (Hewson, 1977). In smaller fields, there was no clear selection for a specific vegetation height by both active and inactive hares, suggesting that vegetation height plays a minor role when field sizes are generally small, and therefore more heterogeneous (Benton et al., 2003).

Hare home ranges were smallest in southern Germany (generally small fields) and largest in northern Germany (generally large fields), indicating that home range size is affected by field sizes (Ullmann et al., 2018). Hares that are potentially excluded from larger fields when vegetation is higher and therefore more dense (Robel, Briggs, Dayton, & Hulbert, 1970) only gain access to high‐quality forage by increasing their home range. This suggests that hares increase their home range size when field sizes are increasing, a finding reported in numerous other studies (Rühe & Hohmann, 2004; Schai‐Braun & Hackländer, 2013; Smith et al., 2004; Tapper & Barnes, 1986). It was suggested that smaller agricultural fields result in a more heterogeneous landscape (Benton et al., 2003), leading to decreased hare home range sizes (Schai‐Braun & Hackländer, 2013), in turn potentially sustaining higher population densities compared to homogenous habitat with large fields as shown in Poland (Panek & Kamieniarz, 1999).

### 4.4 Vegetation height, proximity to field edges, and predation risk

Apart from restricting spatial movements, high vegetation can also reduce the perceptual range of animals. For example, the perceptual ranges of two Neotropical marsupials (*Philander frenatus* and *Didelphis aurita*) were markedly larger in mowed pastures compared to abandoned pastures and manioc (*Manihot esculenta*) plantations (Prevedello, Forero‐Medina, & Vieira, 2011). Higher vegetation potentially decreases the probability of detecting predators, but might at the same time decrease the predation probability (Goheen, Swihart, Gehring, & Miller, 2003). In hares, it was shown that individuals show stronger reactive movements toward simulated predators in short vegetation (Weterings et al., 2016), suggesting that they have an increased risk of being detected by predators. However, the greater visibility in open landscapes might also increase the probability of detecting a predator, and the chances of escape. It was previously reported that hares generally select for proximity to field edges (Petrovan et al., 2013; Schai‐Braun & Hackländer, 2013). Here, we argue that this pattern depends on vegetation height. Both active and inactive hares stayed further from field edges when vegetation was low (<25 cm), possibly to increase the probability to detect and outrun predators. Conversely, they stayed close to field edges in >25 cm high vegetation. Predators generally use field edges more frequently than field centers (e.g., in wildflower strips: (Hummel, Meyer, Hackländer, & Weber, 2017)), which could lead to an increased predation risk close to field edges. Thus, when vegetation is short, both active and inactive hares might remain further from field edges to avoid detection by predators. When vegetation is higher, this might be unnecessary, because predator detection probability is decreased in higher vegetation (Goheen et al., 2003). Additionally, as mentioned above, high vegetation could act as a physical barrier and decrease forage quality. Consequently, as vegetation height increases, hares might remain closer to field edges where they have access to better quality forage (wild herbs and weeds) (Meichtry‐Stier, Jenny, Zellweger‐Fischer, & Birrer, 2014).

### 4.5 The role of vegetation type

Cultivated crops dominate food availability and use by hares in arable landscapes (Reichlin et al., 2006; Schai‐Braun et al., 2015). Overall, active hares selected most vegetation types (bare ground, fabaceae, sugar beet, fallow, maize, and pasture) over cereals, the most common crop type, which was avoided. This indicates that more heterogeneous vegetation types are favorable for hares. Similarly, Tapper and Barnes (1986) reported that hares in England selected areas with various vegetation types and that autumn hare density was positively related to landscape diversity, and an agent‐based modeling approach revealed that hare density increased with habitat heterogeneity (Topping, Høye, & Olesen, 2010).

Concerning inactive hares, we found that fabaceae, fallow, and maize were selected as resting places, the latter two also reported by Bertolino, Montezemolo, and Perrone (2011). Especially, fabaceae and fallow probably provided both cover and forage for inactive hares. Conversely to our prediction, inactive hares avoided higher (>50 cm) vegetation, which is also in contrast to other studies (Neumann et al., 2012; Tapper & Barnes, 1986). However, the vegetation types included in our study were exclusively agricultural, often brassicaceae and cereals, and did not include forest or woodland as in other studies (Neumann et al., 2012; Petrovan et al., 2013; Tapper & Barnes, 1986), which was likely the reason for these different findings. In structurally simple areas with large fields (like northern Germany), hares presumably are not able to include wooded patches in their home range, and thus, select for resting spots in short vegetation away from field edges, allowing them to detect predators from greater distances.

## 5 CONCLUSIONS

Arable fields dominate agricultural land in many European countries, thereby forming the main habitat of hares. We could show that vegetation height is a useful parameter to describe within‐home‐range habitat selection in highly variable landscapes. Hares avoided higher vegetation (>50 cm) probably, because it does not provide high‐quality forage and restricts their spatial movements. Within‐home‐range habitat selection also depended on differences in field sizes and potentially the number of cultivated crops among the three study areas. Both active and inactive hares avoided large fields when vegetation was >50 cm high, leading to larger individual home ranges in these areas. Generally, agricultural intensification has led to increased field sizes and a reduction in field margins (noncropped farmland, such as vegetated paths, shrubland, and wildflower strips) throughout Europe, which likely is the ultimate cause for declining hare and farmland bird populations (Benton et al., 2003; Meichtry‐Stier et al., 2014). Field margins play an important role to preserve biodiversity in agricultural landscapes, because they provide high‐quality forage and shelter throughout the year (Marshall & Moonen, 2002; Meichtry‐Stier et al., 2014; Petrovan et al., 2013). Thus, in order to increase hare numbers in arable landscapes, managers should focus on the improvement of forage quality throughout the year and the reduction of homogenous landscapes. This could be achieved by increasing ecological compensation areas with high structural diversity, like wildflower fields (Meichtry‐Stier et al., 2014). Between 1992 and 2007, the Common Agricultural Policy by the EU made it compulsory for large arable farmers to transform 10% of the agriculturally used land as set‐aside, leading to a partial increase in insect, bird, and mammal numbers (Oppermann, Neumann, & Huber, 2008). We argue that the re‐introduction of mandatory permanent set‐asides as suggested by Langhammer, Grimm, Pütz, and Topping (2017), the reduction in field sizes, for example, via subsidizing small‐scale agriculture, and the farming of various cultivated crop types on a local scale could improve the habitat for hares and other farmland species, halting their decline.

## AUTHORS' CONTRIBUTIONS

MM, WU, CF, PS, and NB developed the design of the work; WU and CF contributed to the data collection; MM and WU prepared the data for the analyses; MM performed the statistical analyses and wrote the manuscript; and WU, CF, PS, and NB commented and improved the manuscript. The authors declare no competing financial interests.

## DATA ACCESSIBILITY

GPS data are deposited in Movebank (https://www.movebank.org/panel_embedded_movebank_webapp?gwt_fragment=page=studies,path=study4048590).
