# Supplementary material for: Habitat selection by the European hare in arable landscapes: The importance of small‐scale habitat structure for conservation
Source: Ecol Evol. 2018 Nov 13;8(23):11619–33. doi: 10.1002/ece3.4613 (PMC6303708; doi:10.1002/ece3.4613)
Supplement: Supplementary file 1 [file ECE3-8-11619-s001.docx]

**Supplementary figures**


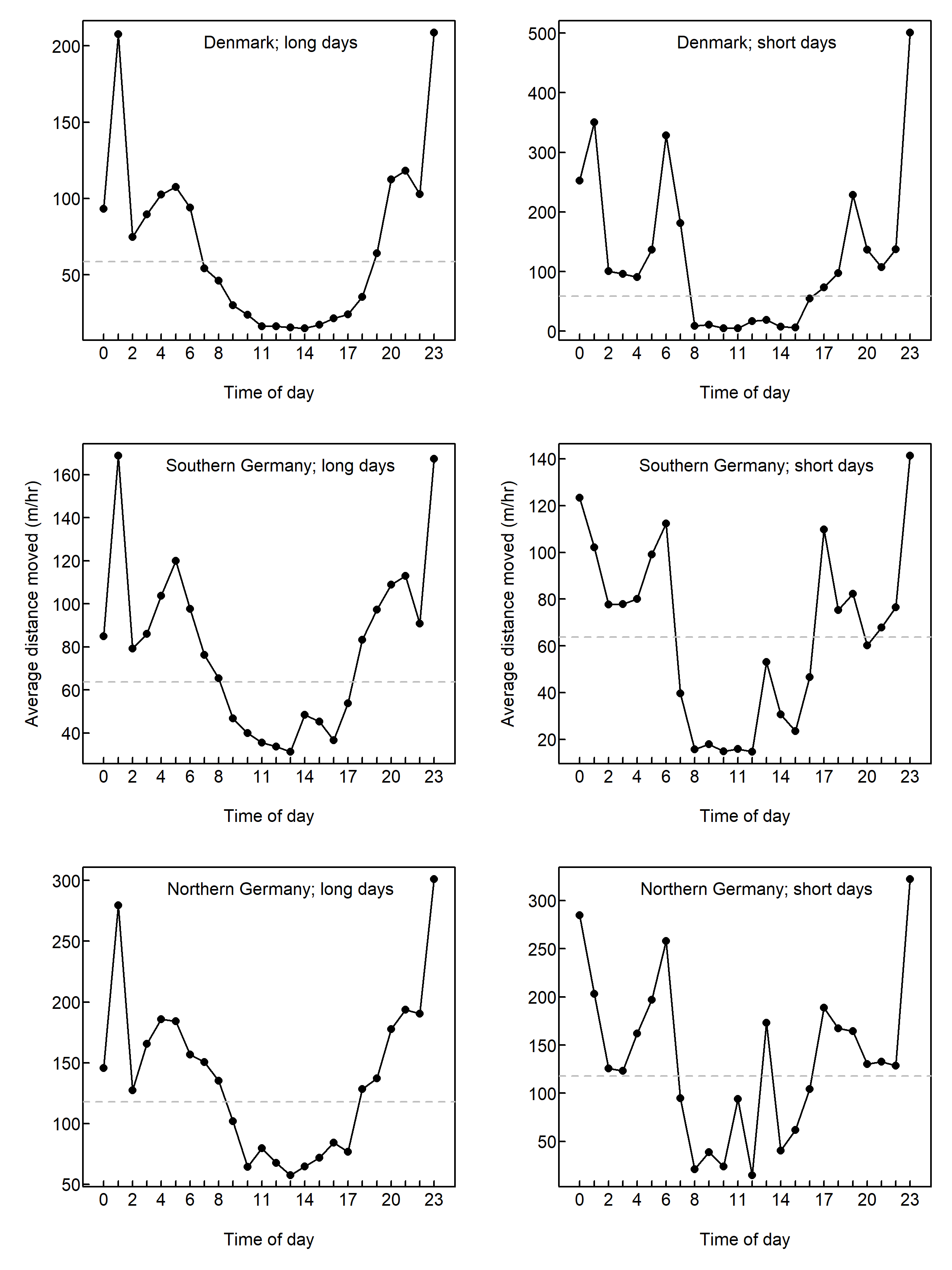


Fig. S1: Plots showing the average distance moved per hour (black dots) as measure of activity separately for long days (>12 hours of daylight; left) and short days (<12 hours of daylight; right) and separately for Denmark (top), Southern Germany (middle) and Northern Germany (bottom). The threshold for activity was set as 75% of the overall average distance moved (grey dashed line), that is, we categorized hares as ‘active’ if hourly distance moved was >75% of the average distance moved, and ‘inactive’ if it was <75% of the average distance moved.
