# Supplementary material for: Habitat selection by the European hare in arable landscapes: The importance of small‐scale habitat structure for conservation
Source: Ecol Evol. 2018 Nov 13;8(23):11619–33. doi: 10.1002/ece3.4613 (PMC6303708; doi:10.1002/ece3.4613)
Supplement: Supplementary file 2 [file ECE3-8-11619-s002.doc]

**Supplementary material**

Table S1: The 20 candidate models for the analyses of habitat selection by European hares (*Lepus europaeus*) separately for active and inactive GPS positions, ranked by AIC_c_. Hare ID, area, and month nested within year were included as random intercept in all models. Hare GPS data were obtained in 2014-2015.

| *Active GPS positions* |  |  |  |  |  |
| --- | --- | --- | --- | --- | --- |
| Model | df | logLik | AIC_c_ | deltaAIC_c_ | AIC_c_ weight |
| Vegetation type + log(Field size) + Vegetation height + log(Edge distance) + log(Edge distance)^2 + Vegetation height x log(Field size) + Vegetation height x log(Edge distance) | 27 | -81245 | 162544 | 0 | 1 |
| Vegetation type + log(Field size) + Vegetation height + log(Edge distance) + log(Edge distance)^2 + Vegetation height x log(Edge distance) | 24 | -81356 | 162760 | 217 | 0 |
| Vegetation type + log(Field size) + Vegetation height + log(Edge distance) + log(Edge distance)^2 + Vegetation height x log(Field size) | 24 | -81361 | 162769 | 226 | 0 |
| Vegetation type + log(Field size) + Vegetation height + log(Edge distance) + log(Edge distance)^2 | 21 | -81566 | 163174 | 631 | 0 |
| Vegetation type + Vegetation height + log(Edge distance) + log(Edge distance)^2 | 20 | -81651 | 163342 | 798 | 0 |
| Vegetation type + log(Field size) + Vegetation height | 19 | -81670 | 163378 | 835 | 0 |
| Vegetation type + Vegetation height | 18 | -81812 | 163660 | 1116 | 0 |
| log(Field size) + Vegetation height + Vegetation height x log(Field size) | 12 | -81881 | 163787 | 1243 | 0 |
| log(Field size) + Vegetation height + log(Edge distance) + log(Edge distance)^2 | 11 | -81931 | 163884 | 1341 | 0 |
| Vegetation height + log(Edge distance) + Vegetation height x log(Edge distance) | 12 | -81947 | 163919 | 1375 | 0 |
| Vegetation height + log(Edge distance) + log(Edge distance)^2 | 10 | -82026 | 164072 | 1528 | 0 |
| log(Field size) + Vegetation height | 9 | -82039 | 164097 | 1553 | 0 |
| Vegetation height | 8 | -82205 | 164427 | 1883 | 0 |
| Vegetation type + log(Field size) + log(Edge distance) + log(Edge distance)^2 | 18 | -82400 | 164836 | 2293 | 0 |
| Vegetation type + log(Edge distance) + log(Edge distance)^2 | 17 | -82466 | 164967 | 2423 | 0 |
| Vegetation type + log(Field size) | 16 | -82501 | 165034 | 2491 | 0 |
| Vegetation type | 15 | -82619 | 165268 | 2724 | 0 |
| log(Field size) + log(Edge distance) + log(Edge distance)^2 | 8 | -82976 | 165968 | 3424 | 0 |
| log(Field size) | 6 | -83096 | 166205 | 3661 | 0 |
| log(Edge distance) + log(Edge distance)^2 | 7 | -83165 | 166344 | 3800 | 0 |
|  |  |  |  |  |  |
| *Inactive GPS positions* |  |  |  |  |  |
| Model | df | logLik | AIC_c_ | deltaAIC_c_ | AIC_c_ weight |
| Vegetation type + log(Field size) + Vegetation height + log(Edge distance) + log(Edge distance)^2 + Vegetation height x log(Field size) + Vegetation height x log(Edge distance) | 27 | -28299 | 56653 | 0 | 1 |
| Vegetation type + log(Field size) + Vegetation height + log(Edge distance) + log(Edge distance)^2 + Vegetation height x log(Edge distance) | 24 | -28318 | 56683 | 31 | 0 |
| Vegetation type + log(Field size) + Vegetation height + log(Edge distance) + log(Edge distance)^2 + Vegetation height x log(Field size) | 24 | -28359 | 56766 | 113 | 0 |
| Vegetation type + log(Field size) + Vegetation height | 19 | -28377 | 56792 | 139 | 0 |
| Vegetation type + log(Field size) + Vegetation height + log(Edge distance) + log(Edge distance)^2 | 21 | -28376 | 56794 | 141 | 0 |
| Vegetation type + Vegetation height | 18 | -28382 | 56800 | 148 | 0 |
| Vegetation type + Vegetation height + log(Edge distance) + log(Edge distance)^2 | 20 | -28382 | 56804 | 151 | 0 |
| Vegetation type + log(Field size) | 16 | -28550 | 57133 | 480 | 0 |
| Vegetation type + log(Field size) + log(Edge distance) + log(Edge distance)^2 | 18 | -28550 | 57135 | 482 | 0 |
| Vegetation type | 15 | -28554 | 57139 | 486 | 0 |
| Vegetation type + log(Edge distance) + log(Edge distance)^2 | 17 | -28554 | 57142 | 490 | 0 |
| Vegetation height + log(Edge distance) + Vegetation height x log(Edge distance) | 12 | -28889 | 57802 | 1150 | 0 |
| log(Field size) + Vegetation height + Vegetation height x log(Field size) | 12 | -28921 | 57866 | 1213 | 0 |
| log(Field size) + Vegetation height | 9 | -28927 | 57872 | 1219 | 0 |
| log(Field size) + Vegetation height + log(Edge distance) + log(Edge distance)^2 | 11 | -28926 | 57874 | 1221 | 0 |
| Vegetation height | 8 | -28933 | 57882 | 1229 | 0 |
| Vegetation height + log(Edge distance) + log(Edge distance)^2 | 10 | -28932 | 57884 | 1231 | 0 |
| log(Field size) | 6 | -29143 | 58298 | 1645 | 0 |
| log(Field size) + log(Edge distance) + log(Edge distance)^2 | 8 | -29142 | 58300 | 1647 | 0 |
| log(Edge distance) + log(Edge distance)^2 | 7 | -29160 | 58335 | 1682 | 0 |
